# *Wolbachia* infection in wild mosquitoes (Diptera: Culicidae): implications for transmission modes and host-endosymbiont associations in Singapore

**DOI:** 10.1186/s13071-020-04466-8

**Published:** 2020-12-09

**Authors:** Huicong Ding, Huiqing Yeo, Nalini Puniamoorthy

**Affiliations:** Department of Biological Sciences, National University of Singapore, 16 Science Drive 4, Singapore, 117558 Singapore

**Keywords:** *Wolbachia*, *Wolbachia* surface protein gene, Reproductive endosymbiont, Tissue-specific polymerase chain reaction, Transmission modes, Host-endosymbiont association

## Abstract

**Background:**

*Wolbachia* are intracellular bacterial endosymbionts found in most insect lineages. In mosquitoes, the influence of these endosymbionts on host reproduction and arboviral transmission has spurred numerous studies aimed at using *Wolbachia* infection as a vector control technique. However, there are several knowledge gaps in the literature and little is known about natural *Wolbachia* infection across species, their transmission modes, or associations between various *Wolbachia* lineages and their hosts. This study aims to address these gaps by exploring mosquito-*Wolbachia* associations and their evolutionary implications.

**Methods:**

We conducted tissue-specific polymerase chain reaction screening for *Wolbachia* infection in the leg, gut and reproductive tissues of wild mosquitoes from Singapore using the *Wolbachia* surface protein gene (*wsp)* molecular marker. Mosquito-*Wolbachia* associations were explored using three methods—tanglegram, distance-based, and event-based methods—and by inferred instances of vertical transmission and host shifts.

**Results:**

Adult mosquitoes (271 specimens) representing 14 genera and 40 species were screened for *Wolbachia*. Overall, 21 species (51.2%) were found positive for *Wolbachia*, including five in the genus *Aedes* and five in the genus *Culex*. To our knowledge, *Wolbachia* infections have not been previously reported in seven of these 21 species: *Aedes* nr. *fumidus*, *Aedes annandalei*, *Uranotaenia obscura*, *Uranotaenia trilineata*, *Verrallina butleri*, *Verrallina* sp. and *Zeugnomyia gracilis*. *Wolbachia* were predominantly detected in the reproductive tissues, which is an indication of vertical transmission. However, *Wolbachia* infection rates varied widely within a mosquito host species. There was no clear signal of cophylogeny between the mosquito hosts and the 12 putative *Wolbachia* strains observed in this study. Host shift events were also observed.

**Conclusions:**

Our results suggest that the mosquito-*Wolbachia* relationship is complex and that combinations of transmission modes and multiple evolutionary events likely explain the observed distribution of *Wolbachia* diversity across mosquito hosts. These findings have implications for a better understanding of the diversity and ecology of *Wolbachia* and for their utility as biocontrol agents.

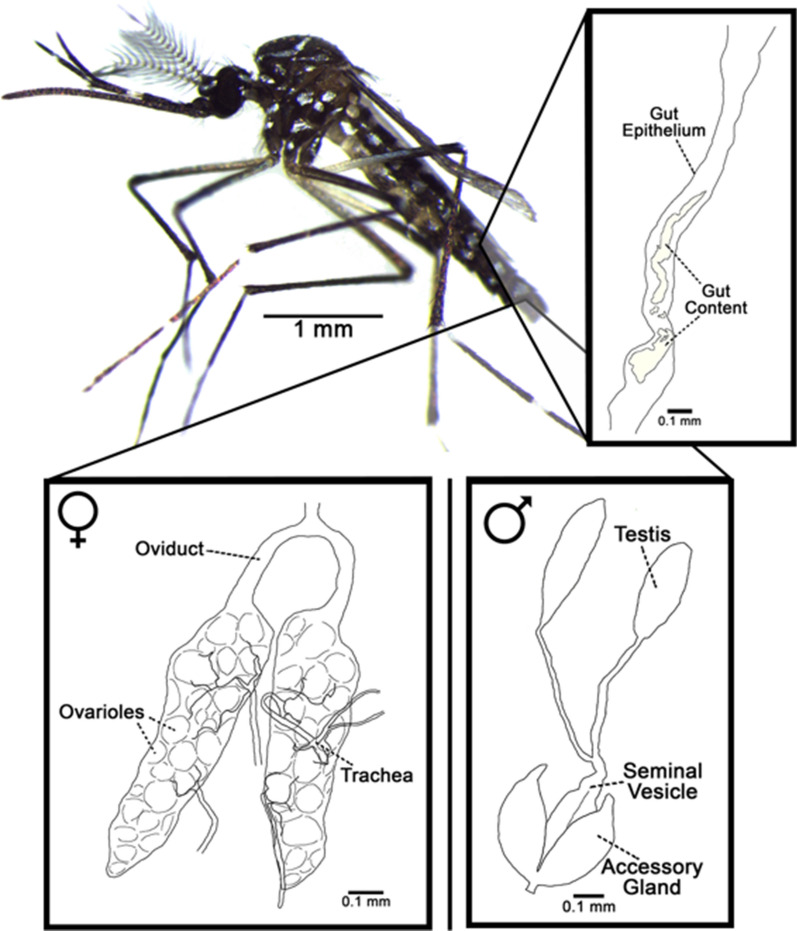

## Background

*Wolbachia* are intracellular endosymbiotic bacteria that alter host reproduction [1]. They are widespread in arthropods, infecting a wide range of insect, crustacean, and nematode species [2, 3]. In some cases, *Wolbachia* exist in a mutualistic relationship with their hosts [4–6]. However, *Wolbachia* are most often recognised as reproductive manipulators that bias the sex ratio of the host offspring towards the production of more infected females [7, 8]. This reproductive manipulation is commonly achieved through four phenotypes—male killing [9], feminisation [10, 11], parthenogenesis [12, 13], and cytoplasmic incompatibility [14, 15]—which increase the endosymbiont’s reproductive success [16]. Owing to their strong influence on host reproduction, an increasing amount of research is being dedicated to exploring the impacts of reproductive endosymbionts on host population dynamics and evolution [17, 18], especially in medically important insects such as mosquitoes. The promising use of *Wolbachia* to alter both mosquito reproduction [19] and arboviral transmission [20] has prompted the deployment of novel *Wolbachia*-infected mosquitoes for population replacement and suppression [21].

Several countries, including Singapore, have started to employ *Wolbachia* as biocontrol agents of mosquitoes by releasing infected mosquitoes [22–24]. However, the presence of naturally occurring endosymbionts in wild mosquito populations has not been adequately assessed. The release of mosquitoes artificially infected with *Wolbachia* might have a profound impact on closely interacting wild mosquito populations through various transmission modes. For instance, horizontal transmission of an introduced *Wolbachia* strain may result in manipulation of the reproductive biology of non-target species, which could potentially result in an unintentional population crash, opening up niches for other vector species [25]. Another possible effect of this type of biocontrol method is the increased likelihood of co-infections with other naturally occurring *Wolbachia* strains or other endosymbionts, such as *Cardinium*, *Rickettsia*, and *Spiroplasma.* These co-infections may result in a synergistic effect on mosquito host fitness and future transmission of endosymbionts [26–29]. Without a detailed characterisation of *Wolbachia* prevalence and diversity among wild mosquitoes, the ecological risk of releasing artificially infected mosquitoes might be overlooked. Therefore, bearing the precautionary principle in mind, it is important to investigate the natural occurrences of *Wolbachia*.

There is also a need to discern the main mode of infection transmission among mosquitoes. Although *Wolbachia* are mainly thought to be vertically transmitted [15, 30], there have been accounts of horizontal transmissions into wild populations through parasitism [31, 32], or through proximity to infected individuals [33]. *Wolbachia* may not be strictly localised in germline tissues, as they have also been detected in somatic tissues such as the gastrointestinal tract and haemolymph [34–36]. The detection of *Wolbachia* in the gastrointestinal tract suggests that they could be horizontally transmitted through uptake from the environment or host sharing [34, 37, 38], whereas their detection in non-gastrointestinal somatic tissues, such as those of jointed appendages, could indicate horizontal bacterial genome integration into the host genome [36]. Currently, detection of *Wolbachia* in mosquitoes is mostly achieved through conventional polymerase chain reaction (PCR) methods using DNA extracted from an entire individual or its abdomen [39–47]. This limits our ability to identify the site of endosymbiont infection within an individual (tissue tropism). Tissue-specific screening of *Wolbachia* is necessary to provide insights and infer the extent of vertical and horizontal transmission.

It has been proposed that host mitochondrial DNA (mtDNA) and *Wolbachia* are maternally co-transmitted within the cytoplasm [17, 48], which suggests a congruency between host mtDNA and *Wolbachia* phylogenies—a consequence of cytoplasmic hitchhiking driven by endosymbiont transmission [17]. In insect systems such as bedbugs where vertical transmission has been established to be the main mode of transmission, *Wolbachia* exhibit clear patterns of cophylogeny with their hosts, with few instances of host shifting or multiple infections within a single host species [49, 50]. In contrast, cophylogeny is not apparent among nematodes and bees, and numerous acquisitions of *Wolbachia* infections through horizontal transmission as well as losses have been shown in these diversified host lineages [51, 52]. The modes of *Wolbachia* transmission among mosquitoes have not been well established, nor has the extent of multiple infections within mosquito hosts or host shifting of these bacteria.

There is presently no comprehensive analysis of the evolutionary associations between *Wolbachia* and their mosquito host species. An understanding of host-endosymbiont associations will not only further our ability to discern the mode of transmission which influences *Wolbachia* diversity, but will also allow for an evaluation of *Wolbachia* host specificity, speciation, and their ability to establish in new hosts. All of this is key to understanding the diversity and ecology of *Wolbachia*, and their utility in biocontrol methods.

This study has three major research objectives. First, to examine the prevalence and diversity of *Wolbachia* among wild mosquitoes from Singapore. Second, to determine the tissue tropism of *Wolbachia* infection in mosquitoes using a tissue-specific PCR screening method. Finally, to reconstruct the evolutionary associations between *Wolbachia* and their mosquito hosts to provide a basis for an understanding of host-endosymbiont evolution.

## Methods

### Adult mosquito collection and identification

**Fig. 1a–d:**
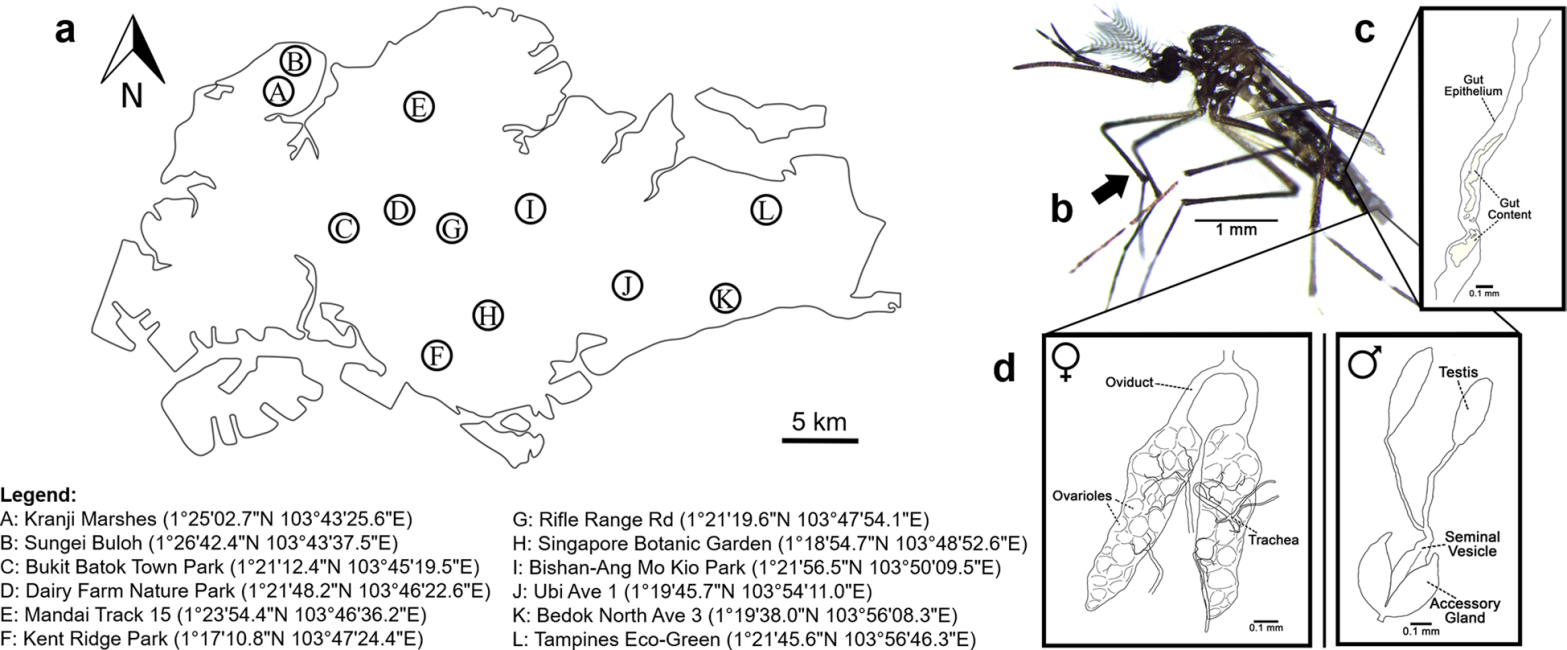
Map of sampling sites and diagrammatic images of *Aedes aegypti* and its dissected tissues. **a** Various mosquito collection localities across Singapore and their respective coordinates, **b** mosquito leg, **c** gut, **d** female reproductive tissue (*left*) and male reproductive tissue (*right*)

Mosquito samples were collected from 12 localities across Singapore between March 2018 and November 2019 (Fig. 1a). Three methods were employed to collect the samples: CO_2_-baited Centers for Disease Control and Prevention traps, sweep-netting using hand-held fan traps, and larval sampling [53]. For the latter, dipping was carried out at streams and ponds and pipettes were used to collect larvae from various microhabitats, including tree holes, plant axils, and artificial containers. Thereafter, the field-collected larvae were reared to adults in an incubator maintained at 26 °C and 70% relative humidity, under a 12:12-h (day:night) photoperiod. Larvae were fed with pulverised fish food (TetraMin Granules) daily. Mosquitoes were identified using relevant taxonomic keys and descriptions [54–59]. A subset of individuals from commonly sampled species was selected and preserved in phosphate-buffered saline solution at – 80 °C for subsequent dissection step.

### Tissue-specific dissection

Tissue-specific dissection was carried out on each adult mosquito sample to isolate the leg, gut, and reproductive tissues (Fig. 1b–d). To prevent the contamination of tissues with bacteria on the external surface of the mosquito, the leg was removed first before isolating the gut and reproductive tissues. All dissection equipment and microscope slides were thoroughly wiped with 70% ethanol before commencing dissection of the next sample. Dissected tissues were individually placed into a 96-well plate on ice to prevent DNA degradation.

### DNA extraction, PCR amplification, and sequencing

DNA extraction of each dissected tissue was performed using 7 μl of QuickExtract DNA Extraction Solution (Lucigen, Madison, USA) in a thermocycler (Eppendorf, Hamburg, Germany) with the following protocol: 65 °C for 18 min, followed by 98 °C for 2 min, ending with cooling on ice for at least 10 min. All dissected tissues were screened for *Wolbachia* infections following single-primer PCR protocols described by Martin et al. [26] with slight modifications to the cycle conditions. The *Wolbachia* surface protein gene (*wsp*) general primers, wsp81F (5′-TGGTCCAATAAGTGATGAAGAAACTAGCT-3′) and wsp691R (5′-AAAAATTAAACGCTACTCCAGCTTCTGCAC-3′), were used in this study [60]. In addition, a fragment of the cytochrome c oxidase subunit I (*cox*1) gene of the mosquito hosts was also amplified using primers LCO1498 (5′-GGTCAACAAATCATAAAGATATTGG-3′) and HCO2198 (5′-TAAACTTCAGGGTGACCAAAAAATCA-3′) [61]. This served to confirm host identity and acted as an internal control. We used DNA from known *Wolbachia*-infected *Nasonia* specimens as positive controls for this study.

All PCR procedures were performed in reaction mixtures consisting of 12.5 μl of GoTaq G2 Green Mastermix (Promega, Madison, USA), 1 μl of 1 mg ml^−1^ bovine serum albumin, 0.184 μl of 25 mM magnesium chloride, 1.5 μl of extracted DNA, and 1.5 μl each of 5 μM *wsp* forward and reverse primers for *Wolbachia* PCR screens or 1.0 μl each of 5 μM LCO1498 and HCO2198 primers for *cox*1 PCRs. Double-distilled water was used to top up the reaction mixture to a final volume of 25 μl. PCR amplification of positive and negative controls was also conducted simultaneously.

PCR conditions were as follow: 94 °C for 5 min, followed by 35 cycles of 95 °C for 30s, 55 °C for 45s, and 72 °C for 1 min, with a final elongation step of 72 °C for 10 min. Amplicons were separated by gel electrophoresis on 2% agarose gel stained with GelRed (Biotium, Fremont, USA) and visualised under a ultraviolet transilluminator (Syngene, Cambridge, UK). PCR products were purified using SureClean Plus (Bioline, London, UK) following the manufacturer's protocol. Samples were sequenced by First Base Laboratories (Axil Scientific, Singapore), using a 3730XL DNA Analyzer (Applied Biosystems, Waltham, USA). Obtained sequences were edited and aligned using Geneious Prime (version 2019.2.3) (https://geneious.com). Similarities with publicly available sequences were assessed using the Basic Local Alignment Search Tool (BLAST) [62].

### Statistical analyses

To test if there were significant differences in *Wolbachia* infection across the different mosquito tissues, Cochran’s Q-test was carried out. As a follow-up, McNemar’s post hoc test was employed to identify the tissue pairs that differed significantly in infection. Individuals for which the internal control (*cox*1 gene) was not amplified successfully for any of the three dissected tissues were excluded from this statistical analysis. The effect of sex on host infection was also tested using binary logistics regression with sex as a categorical dependent variable and infection outcome as a binary independent variable. Logistic regression was conducted on a subset that only included species that had a roughly similar representation of both sexes, i.e. for every species included, the number of individuals of the less common sex was proportionally at least 60% of the number of individuals of the more common sex. This was to prevent a biased analysis due to a dataset with unequal representation of the sexes. Statistical significance was determined as* P* < 0.05. All statistical analyses were performed in R version 3.6.2 [63] with packages nonpar [64], rcompanion [65], and ISLR [66].

### Sequence analyses

Multiple alignment of consensus sequences was carried out using the ClustalW algorithm with default settings (gap penalty = 15, gap extension penalty = 6.66) [67], in software MEGA X [68]. Mosquito *cox*1 sequences generated in this study were aligned with 61 reference *cox*1 barcodes of identified local mosquitoes from Chan et al. [53]. For *wsp* sequences, the generated sequences were aligned with 54 available *wsp* sequences of known *Wolbachia* strains obtained from GenBank [69]. Short sequence reads (< 500 base pairs) were excluded.

Neighbour-joining (NJ) phylogenetic trees for mosquito hosts and *Wolbachia* were reconstructed using the sequenced *cox*1 gene fragment and the *wsp* gene, respectively. *cox*1 sequences from previous publications were not included because a comparison of the genetic relationships between the hosts was not the aim of this research. Instead, 54 *wsp* sequences from GenBank were included in the construction of the *Wolbachia* NJ tree. The NJ tree reconstruction was performed with the Kimura two-parameter model as the nucleotide substitution model in MEGA X [68]. Internal gaps were treated as indels and terminal gaps as missing for *wsp* sequences. Bootstrap probabilities were estimated by generating 1000 bootstrap replicates. We designated two biting midge species, *Culicoides asiana* (KJ162955.1) and *Culicoides wadai* (KT352425.1), as outgroups for the host NJ tree construction. Due to the lack of an appropriate endosymbiont outgroup [51], the *Wolbachia* NJ tree was midpoint rooted.

When possible, *Wolbachia* strains were classified into supergroups and putative strains using 97% bootstrap probability as a threshold [60]. *Wolbachia* surface protein sequences that did not have 97% bootstrap support were evaluated on a case-by-case basis. For example, sequences which clustered closely together and had a relatively high support value (> 90%) were deemed as originating from the same putative strain.

Putative strains which were infectious to only one host species were categorized as ‘specialists’ and those which infected two or more hosts as ‘generalists’. Then, the standardised phylogenetic host specificity (SPS) score of each generalist strain was calculated by adapting the method outlined by Poulin et al. [70] and Kembel et al. [71]. SPS measures the degree of phylogenetic relatedness among host species infected by the same endosymbiont strain. It also tests for significance by comparing it with null models generated with 999 replicates of random host-endosymbiont associations. A positive SPS value with a high *P-*value (*P* > 0.95) indicates a high degree of host flexibility where *Wolbachia* infect hosts which are phylogenetically even. A negative SPS value with low* P*-value (*P* < 0.05) suggests a low degree of host flexibility where the infected hosts are phylogenetically clustered together. SPS scores were calculated using R package picante [71].

### Evolutionary analyses of the mosquito-*Wolbachia* relationship

Three distinct methods were used to explore the evolutionary associations between mosquito hosts and their *Wolbachia* endosymbionts. The analyses were carried out using pruned phylogenies where each species is represented by a single individual.

First, using the software TreeMap 3.0 [72], a tanglegram was created between host and endosymbiont NJ trees to visualise mosquito-*Wolbachia* associations. A tanglegram is useful as a pictorial representation of the interactions between two phylogenies [73]. TreeMap also seeks to minimise the entanglement between the two trees to provide a clearer visualisation of the phylogenetic relationship between host and endosymbiont [72].

Second, ParaFit Global test, a distance-based method, was employed to quantitatively estimate congruence between the host and endosymbiont phylogenetic trees by comparing genetic distances among infected host species and the *Wolbachia* strains [74]. The null hypothesis for this test states that the associations between host and endosymbiont trees are random, whereas the alternative hypothesis suggests that there are strong associations between hosts and parasites, which are indicated by phylogenetic distances. Significance was tested by comparing the observed associations between host and endosymbiont with randomised associations generated with 5000 permutations. The respective host-endosymbiont associations which contributed significantly to the ParaFit Global statistics were also identified by performing a Parafit Link test. ParaFit tests were performed with the Cailliez correction to correct for negative eigenvalues generated [75] using R package ape [76].

Third, an event-based analysis was performed in Jane 4.0 [77] to map out potential evolutionary events of the endosymbiont in relation to the host phylogeny [78]. Five evolutionary events were considered: co-speciation (host and endosymbiont speciate simultaneously), duplication (intra-host speciation), duplication with host shift (endosymbiont host shifts), loss (host speciates but endosymbiont fails to establish in one of the new lineages), failure to diverge (host speciates and endosymbiont remains in both lineages). As each event is expected to have differing likelihoods, default cost values were attached to each of the events. Jane 4.0 determined the best reconstruction of evolutionary events by minimising the overall cost. The following cost-scheme regime was used with 100 generations and a population size of 300: co-speciation = 0, duplication = 1, duplication with host shift = 2, loss = 1, and failure to diverge = 1 [79]. As a follow-up, random tip mapping (randomisation of host-endosymbiont associations) was carried out for 50 iterations, to determine if the overall cost of reconstruction was significantly lower than expected by chance. If 5% or fewer of the random solutions have costs lower than the reconstructed coevolution phylogeny, there is support for the coevolution of the hosts and endosymbionts through co-speciation.

## Results

### Prevalence of *Wolbachia* in wild-caught mosquitoes

A total of 271 adult mosquitoes, representing 40 species and 14 genera, were collected from 12 localities in Singapore (Fig. 1a). Overall, infection prevalence was moderate with 119 out of 271 (43.9%) individuals screening positive for *Wolbachia* (Table 1). In total, 21 (51.2%) species were positive for *Wolbachia*. According to our knowledge, *Wolbachia* infection in seven of these species is reported here for the first time (Table 1). *Wolbachia* were detected in all genera except for *Aedeomyia*, *Anopheles* and *Mimomyia* (i.e. 11 out of 14 genera; 78.6%). Five out of the seven *Aedes* species collected (71.4%) were positive for *Wolbachia*, while in the genus *Culex*, five out of 16 species (31.3%) were positive. Some of the screened species in the genera *Aedes* and *Culex* that were positive for *Wolbachia*, such as *Aedes albopictus* and *Culex quinquefasciatus*, are medically important vector species.

**Table 1:** Percentage infection of *Wolbachia* in 40 mosquito species collected from 12 Singapore localities

Mosquito species	Localities												Total	Infection (%)	Supergroup
	BN	BA	BB	DF	KR	KJ	M	RR	SBG	SBL	T	U			
*Aedeomyia catastica*	–	0/1	–	–	–	–	–	–	–	–	–	–	0/1	0.0	–
*Aedes aegypti*	0/1	–	–	–	–	–	–	–	–	–	–	0/13	0/14	0.0	–
*Aedes albolineatus*	–	–	–	–	–	–	0/3	–	–	–	–	–	0/3	0.0	–
*Aedes albopictus*	–	–	–	6/10	6/10	3/6	6/11	–	–	–	–	–	21/37	56.8	A, B
*Aedes annandalei*^a^	–	–	–	–	3/4	–	8/9	–	–	–	–	–	11/13	84.6	A
*Aedes* nr. *fumidus*^a^	–	–	–	–	–	–	–	–	–	6/10	–	–	6/10	60.0	A
*Aedes gardnerii*	–	–	–	–	–	–	1/1	–	–	–	–	–	1/1	100.0	A
*Aedes malayensis*	–	–	–	1/2	13/16	0/2	–	–	–	–	–	–	14/20	70.0	A
*Anopheles barbirostris* complex	–	–	–	0/2	–	–	0/2	–	–	–	–	–	0/4	0.0	–
*Anopheles lesteri*	–	–	–	–	–	0/2	–	–	–	–	–	–	0/2	0.0	–
*Anopheles sinensis*	–	0/12	–	–	–	–	–	–	–	–	–	–	0/12	0.0	–
*Armigeres kesseli*	–	–	–	–	3/3	–	–	–	–	–	–	–	3/3	100.0	B
*Coquillettidia crassipes*	–	–	–	2/2	6/7	4/4	–	–	–	–	–	–	12/13	92.3	B
*Culex* (*Lophoceramyia*) spp.^c^	–	–	–	–	0/1	0/2	1/9	–	–	–	0/2	–	1/14	7.1	B
*Culex bitaeniorhynchus*	–	–	–	–	0/1	–	–	–	–	–	–	–	0/1	0.0	–
*Culex brevipalpis*	–	–	–	0/1	–	–	0/2	–	–	–	–	–	0/3	0.0	–
*Culex nigropunctatus*	–	–	–	–	–	0/1	0/2	–	–	–	–	–	0/3	0.0	–
*Culex pseudovishnui*	–	–	–	–	11/12	–	4/4	–	3/5	1/1	–	–	19/22	86.4	B
*Culex quinquefasciatus*	–	5/8	–	–	–	–	–	–	–	–	–	–	5/8	62.5	B
*Culex sitiens*	–	–	–	–	–	–	–	–	–	2/4	–	–	2/4	50.0	B
*Culex* sp.	–	–	–	–	–	–	0/2	–	–	–	–	–	0/2	0.0	–
*Culex tritaeniorhynchus*	–	–	–	–	–	2/5	–	–	–	0/1	0/1	–	2/7	28.6	UC^b^
*Culex vishnui*	–	–	–	–	–	–	0/2	–	–	–	0/3	–	0/5	0.0	–
*Malaya genurostris*	–	–	2/4	–	0/1	4/13	–	–	0/1	–	–	–	6/19	31.6	B
*Mansonia dives*	–	–	–	–	–	–	0/2	–	–	–	–	–	0/2	0.0	–
*Mansonia indiana*	–	–	–	–	–	3/3	–	–	–	–	–	–	3/3	100.0	B
*Mimomyia luzonensis*	–	–	–	–	–	0/1	–	–	–	–	–	–	0/1	0.0	–
*Tripteroides* sp.	–	–	–	–	0/7	–	½	–	–	–	–	–	1/9	11.1	UC^b^
*Uranotaenia obscura*^a^	–	–	–	2/4	–	–	2/2	1/1	–	–	–	–	5/7	71.4	A
*Uranotaenia* sp.	–	–	–	1/2	–	–	–	–	–	–	–	–	1/2	50.0	A
*Uranotaenia trilineata*^a^	–	–	–	–	–	–	1/1	–	–	–	–	–	1/1	100.0	B
*Verrallina butleri*^a^	–	–	–	–	–	1/1	–	–	–	–	–	–	1/1	100.0	UC^b^
*Verrallina* sp.^a^	–	–	–	–	–	–	–	1/5	–	–	–	–	1/5	20.0	UC^b^
*Zeugnomyia gracilis*^a^	–	–	–	1/2	–	–	1/13	1/4	–	–	–	–	3/19	15.8	B
Total	0/1	5/21	2/4	13/25	42/62	17/40	25/67	3/10	3/6	9/16	0/6	0/13	119/271	43.9	

*BN* Bedok North Avenue 3, *BA* Bishan-Ang Mo Kio Park, *BB* Bukit Batok Town Park, *DF* Dairy Farm Nature Park, *KR* Kent Ridge Park, *KJ* Kranji Marshes, *M* Mandai Track 15, *RR* Rifle Range Road, *SBG* Singapore Botanic Garden, *SBL* Sungei-Buloh, *T* Tampines Eco-Green, *U* Ubi Avenue 1

^a^Species in which, according to our knowledge, *Wolbachia* infection has not been previously reported

^b^*Wolbachia* infections that were unclassified (*UC*) with respect to supergroup [60] because their DNA sequences were either too short (< 400 base pairs), or there were alignment issues during the phylogenetic analyses

^c^*Culex* (*Lophoceramyia*) comprises seven unique species, which were not identified here

The infection rates varied across the mosquito species. Notably, there was variation in the percentage of infection between species that are epidemiologically related. For instance, *Wolbachia* infection was not detected in *Aedes aegypti*. However, infection was moderately high (56.8%) for *Aedes albopictus*. There was also a difference in the infection rate of two closely related species, *Culex pseudovishnui* (86.4%) and *Culex vishnui* (0%) [53].

Locality did not seem to play a role in the *Wolbachia* infection of mosquito hosts. Among species that have a wide range across Singapore, the percentage of infection was consistent in populations across different habitats. For example, the infection percentage was consistently high for *Cx. pseudovishnui*, while consistently low for *Malaya genurostris*. Based on our results, species identity was a better predictor of infection status than locality.

Based on a data subset containing 153 individuals (45.8% males) representing 12 mosquito species, sex was a significant explanatory variable, and there was a significantly lower infection prevalence in males than females (odds ratio 0.434; binary logistics regression: *Z* = – 2.48, *df* = 151, *P* = 0.013).

### Tissue tropism of *Wolbachia* infection in mosquitoes

Among the 159 successfully amplified *cox*1 sequences, *Wolbachia* infection was mainly observed in the reproductive tissues. Among the reproductive tissues of 159 dissected individuals, 42.1% (*n* = 67) were infected. Percentage infection was lower in the gut (5.7%, *n* = 9) and leg (3.1%, *n* = 5) tissues. The difference in percentage infection across the three dissected tissues was statistically significant (Cochran’s *Q*-test: *Q* = 109.5, *df* = 2, *P* < 0.0001). The percentage of infection in the reproductive tissues was significantly higher than in the gut (McNemar’s post hoc test: *P* < 0.0001) and leg tissues (McNemar’s post hoc test: *P* < 0.0001), but the difference in percentage of infection between the gut and leg tissues was not significant (McNemar’s post hoc test: *P* = 1.0). Notably, the amplicon size of *wsp* in the gut and leg tissues tended to be shorter than 400 base pairs.

### *Wolbachia* diversity among mosquito fauna from Singapore

**Fig. 2:**
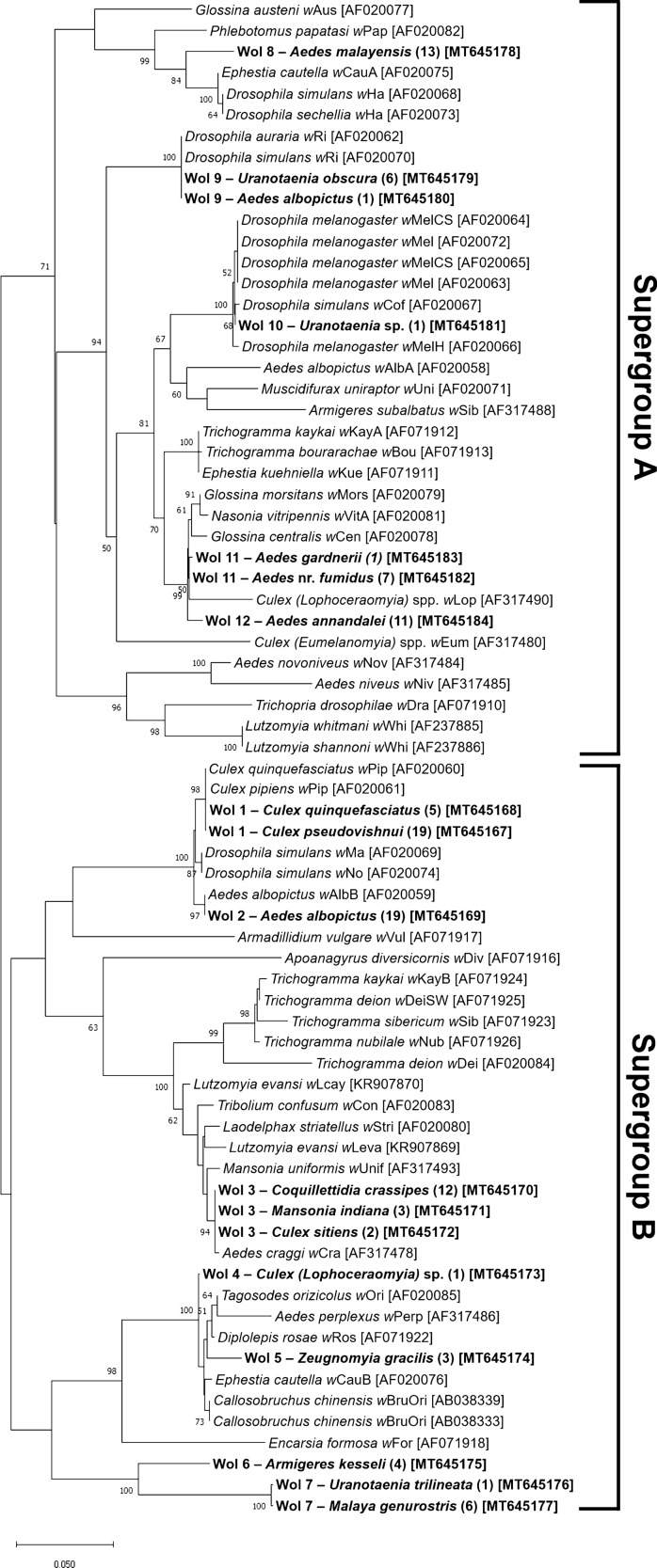
*Wolbachia* neighbour-joining (NJ) tree constructed with the *Wolbachia* surface protein gene (*wsp*). All analysed sequences generated from this study (*bold*) were broadly classified into *Wolbachia* supergroups A or B and clustered into 12 putative strains (‘Wol 1’–‘Wol 12’). The number of sequences of each putative strain is indicated* within parentheses*. Also included are 54 sequences obtained from GenBank. Taxa are labelled as the host from which the *Wolbachia* strain was isolated, followed by the strain name. The NJ tree was mid rooted due to a lack of appropriate outgroups [45]. Bootstrap probability (generated with 1000 replicates) higher than 50% is indicated on the tree. Genbank accession number of each sequence is indicated* within brackets*

Following Zhou et al. [60], all *wsp* sequences obtained in this study can be broadly classified into A and B *Wolbachia* supergroups. Out of 21 infected species, six were infected with supergroup A, ten with supergroup B, and one species, *Ae. albopictus*, was infected with both supergroups (Fig. 2). Infection of the remaining four species (*Culex tritaeniorhynchus*, *Tripteroides* sp., *Verrallina butleri*, and *Verrallina* sp.) was unclassified due to short sequences (< 400 base pairs) or sequence alignment issues during sequences analyses. The analysed *wsp* sequences were also clustered into 12 putative strains: ‘Wol 1’ to ‘Wol 12’. Four (Wol 1, Wol 2, Wol 3, and Wol 8) out of the 12 putative strains could be matched to previously typed strains [60, 80]. *Wolbachia* strains from this study are also closely related to those isolated from other insect groups (Fig. 2). For instance, Wol 9 and Wol 10 are closely related to the *Wolbachia* strains harboured by *Drosophila* spp. (bootstrap value > 99%).

### Host specificity of *Wolbachia* strains

**Fig. 3:**
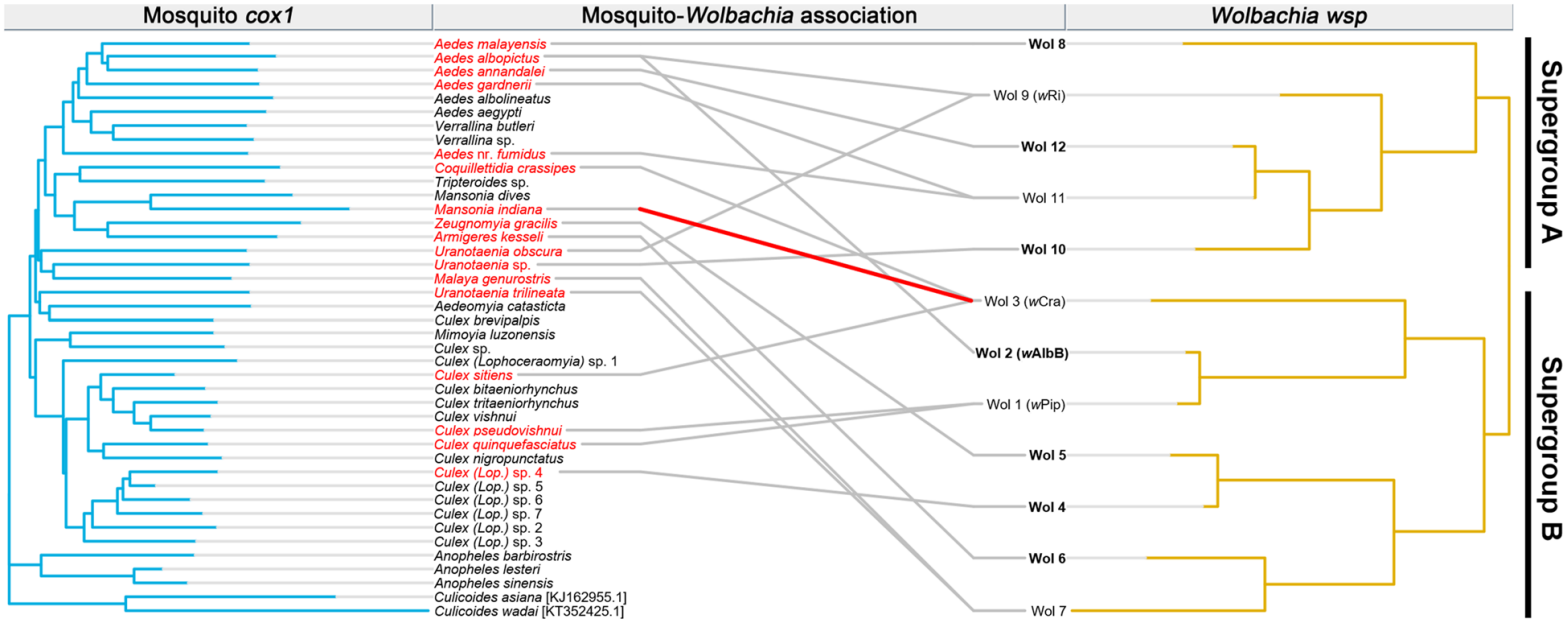
Tanglegram of mosquito *cox*1 NJ tree compared to the *Wolbachia* endosymbiont NJ tree. Mosquito host species that harboured *Wolbachia* infection are indicated in* red*. Specialist *Wolbachia* strains are in* bold*.* Grey lines* represent the associations between hosts and endosymbionts. A* red line* indicates the host-endosymbiont association that was significant in the Global ParaFit test of congruence between host and endosymbiont phylogenies (Parafit Link test: ParaFit Link = 0.045, *P* = 0.029)

The degree of host specificity varied across the 12 putative strains. Seven out of the 12 strains (Wol 2, Wol 4, Wol 5, Wol 6, Wol 8, Wol 10, and Wol 12) were considered as specialists. These strains were host specific and were only detected in one host species each (Fig. 3). The remaining five strains were considered as generalists as they were found in more than one host. Amongst the generalists, Wol 3 was found in the highest number of host species, i.e. three, *Coquillettidia crassipes*, *Mansonia indiana*, and *Culex sitiens*. The SPS scores revealed that Wol 1 had the lowest degree of host flexibility (SPS test: *Z* = – 1.41, *P* = 0.049). Wol 7 had the highest degree of host flexibility, but this was not statistically significant (SPS test: *Z* = 0.07, *P* = 0.779) (Table 2).

**Table 2:** Standardised phylogenetic host-specificity (*SPS*) scores of putative *Wolbachia* generalists

Putative *Wolbachia* strain	No. of infected hosts	Phylogenetic host-specificity score	SPS score	*P*-value
Wol 1	2	0.281	− 1.41	0.049*
Wol 3	3	0.391	− 0.162	0.421
Wol 7	2	0.281	0.068	0.779
Wol 9	2	0.281	− 0.234	0.249
Wol 11	2	0.281	− 0.817	0.157

* P < 0.05

### Evolutionary relationship between mosquitoes and *Wolbachia*

We recorded 18 counts of mosquito-*Wolbachia* associations in wild-caught mosquitoes from Singapore. A visualisation of these associations using a tanglegram showed patterns of broad associations (Fig. 3). For instance, the clade which consists of *Aedes* species was observed to be mostly associated with *Wolbachia* supergroup A. In contrast, other species, especially the clade representing various *Culex* species, had numerous associations with *Wolbachia* supergroup B.

The distance-based quantitative test showed that mosquito and *Wolbachia* phylogenies were weakly congruent at the global level (ParaFit Global test: ParaFit Global = 0.006, *P* = 0.048). Among the numerous host-endosymbiont links, only the association between *Mansonia indiana* and Wol 3 was statistically significant (ParaFit Link test: ParaFit Link = 0.045, *P* = 0.029) (Fig. 3).

**Fig. 4:**
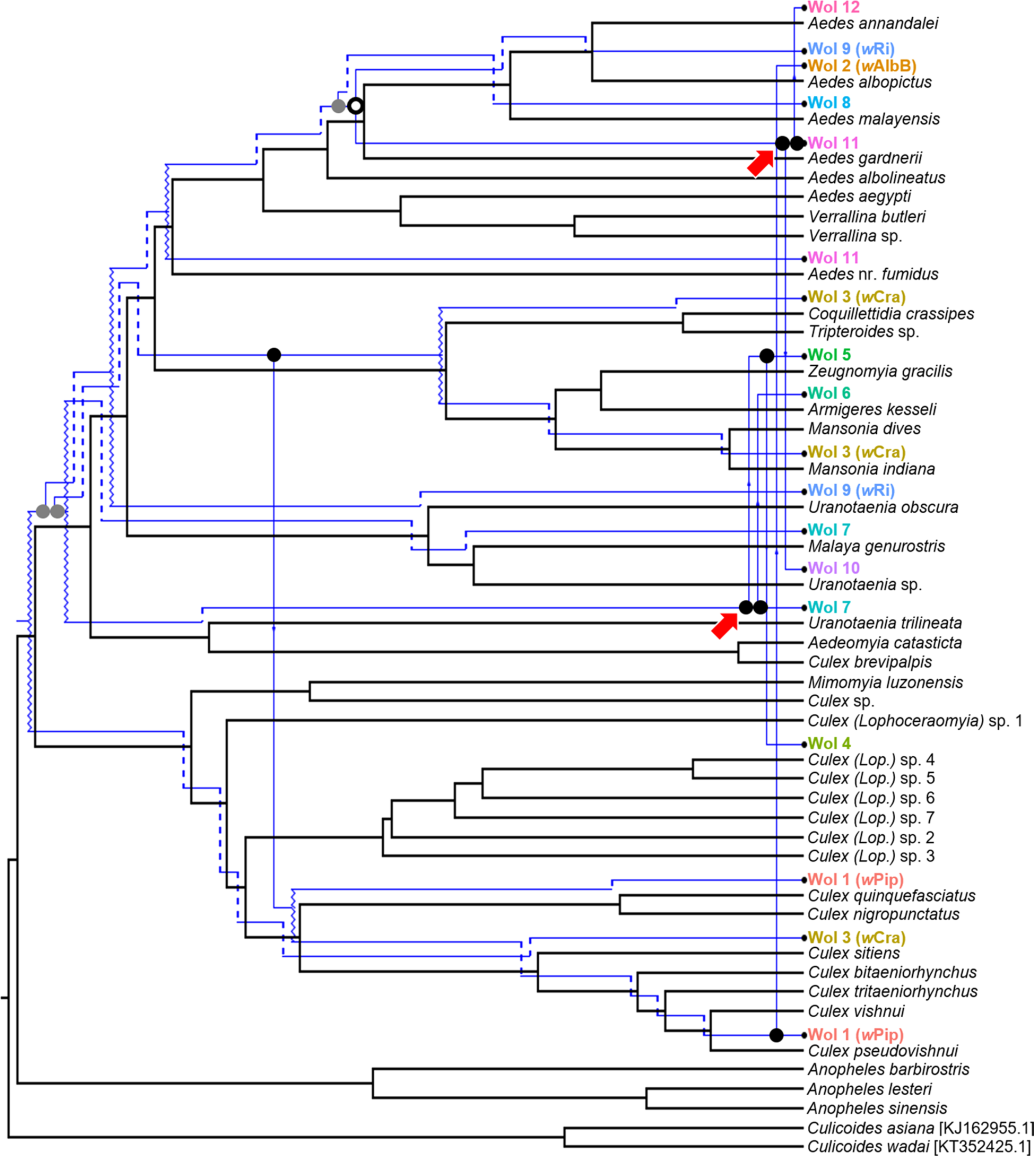
Least-cost evolutionary reconstruction between mosquito (*black*) and *Wolbachia* (*blue*) phylogenies achieved using Jane 4.0. In total, one co-speciation event (*open circle*), three counts of duplication (*grey dot*), seven counts of duplication with host shift (*black dot* with an* arrow* pointing outwards), 29 losses (*dotted line*), and six counts of failure to diverge (*squiggly line*) were mapped out.* Red arrows* indicate periods where multiple host shifts occurred in succession

The event-based analysis between mosquito and *Wolbachia* phylogenies resulted in a reconstructed output of one co-speciation event, three counts of duplication, seven counts of duplication with host shift, 29 losses, and six counts of failure to diverge, amounting to a total cost of 52 (Fig. 4). Interestingly, the number of duplications with a host shift and losses was much greater than co-speciation events. Notably, multiple host shift events tend to follow after loss events occurring earlier in the evolutionary history of the endosymbiont. For example, we see instances of consecutive host shifts to new hosts that were not previously infected (Fig. 4, red arrows). Additionally, based on random tip mapping, 14% of the random solutions had lower costs than the reconstructed output. Overall, there was support for multiple host shift events and losses of *Wolbachia* among the mosquitoes, and no clear signal for mosquito-*Wolbachia* cophylogeny.

## Discussion

### Detection of *Wolbachia* infection and distribution in wild mosquitoes

In this study, the PCR-based *Wolbachia* screening method had a high positive detection rate with 86.3% of all sequenced amplicons having successful BLAST matches to *Wolbachia*. This suggests that the conventional PCR method used is adequate for *Wolbachia* detection. Even if the study had been carried out without the additional DNA sequencing step, observed amplicon bands would likely have indicated true positives.

Our results indicate that *Wolbachia* are widespread across members of the family Culicidae. To our knowledge, *Wolbachia* infections have not been previously reported in seven of the mosquito species collected in this study. Overall, the percentage infection of screened individuals was 43.9%, which was largely congruent with percentages reported in past studies from the Oriental region, i.e. 31% infection in Malaysia [81], 26.4% in Sri Lanka [39], and 61.6% in Thailand [82]. At the species level, previous studies reported *Wolbachia* infection in 40% of all tested mosquito species in India [83], 18.2% in Sri Lanka [39], 51.7% in Taiwan [84], and between 28.1% and 37.8% in Thailand [82, 85]. Our study showed that 51.2% of all tested species were infected with *Wolbachia*, which is generally higher than the percentage reported in most studies. This was likely due to the broad range of species tested, including those from the genera *Malaya*, *Verrallina*, and *Zeugnomyia* [85]. It is also possible that infection prevalence may vary across geographical regions.

*Wolbachia* detection in three medically important mosquito genera, *Culex*, *Anopheles*, and *Aedes*, was highly consistent with that of past studies. These genera are responsible for the transmission of vector-borne diseases such as filariasis, malaria and arboviral diseases [86]. Within the genus *Culex*, *Wolbachia* infection has been reported to be variable across its member species [39, 46, 82, 84]. In this study, infections were observed only in five out of 16 *Culex* species. We noticed moderately high *Wolbachia* infection in *Cx. quinquefasciatus* (62.5%), which is a member of the *Culex pipiens* complex responsible for the transmission of filariasis in Singapore [86, 87]. Surprisingly, no *Wolbachia* infection was observed in *Cx. vishnui*–which has been found to harbour Japanese encephalitis virus in Southeast Asia [89]–although it is closely related to *Cx. pseudovishnui* [88] in which the rate of *Wolbachia* infection was high. However, studies in India and Thailand showed a reverse pattern, with *Wolbachia* infection present in *Cx. vishnui* but not in *Cx. pseudovishnui* [39, 85]. As the two species are morphologically similar [53], DNA barcoding was conducted to aid morphological identification, and thus avoid any misidentification. The results lend further support to possible variation in infection prevalence between geographically distant populations.

We did not detect *Wolbachia* in any of the wild-caught *Anopheles* species (18 individuals representing three species), many of which are potential malaria vectors [86]. This is largely consistent with previous reports from different countries [39, 90, 91]. The absence of *Wolbachia* in *Anopheles* mosquitoes is thought to be due to the unsuitability of *Anopheles* reproductive tissues for *Wolbachia* establishment [84, 85]. However, there have been recent reports of *Wolbachia* detected in wild *Anopheles* mosquitoes from West Africa [42, 92, 93] and Malaysia [94]. Knowledge of natural *Wolbachia* infections in *Anopheles* mosquitoes is important for malaria control strategies [93], hence more wild-caught *Anopheles* samples should be screened in Singapore to determine more accurately their infection status.

*Wolbachia* were not detected in *Ae. aegypti*, the primary vector of dengue in Southeast Asia [87]. Conversely, *Wolbachia* infection was moderately high in the secondary vector *Ae. albopictus*. These results are highly consistent with those of past studies, which reported an absence of infection in wild *Ae. aegypti* [21, 95], but found stable infection in wild *Ae. albopictus* [96]. Although *Ae. aegypti* and *Ae. albopictus* belong to the same subgenus, *Stegomyia*, and occupy similar ecological niches [97], they are rarely found in the same locality, [43, 98, 99], as also observed in this study. This could imply a certain degree of competitive exclusion between the two species, preventing them from occupying the same space. There is evidence that symbionts may influence a host’s resource acquisition and specificity, which may ultimately lead to competitive exclusion between closely related host species with differing symbiont infections [100, 101]. However, research on *Wolbachia*-induced competitive exclusion is scarce except for a few studies on heterogonic gall wasps [102], grasshoppers [103], and gall-inducing aphids [104]. Given the widespread influence of *Wolbachia*, future research should explore potential cases of *Wolbachia*-induced competitive exclusion between closely related species of mosquitoes as this may have major implications for an understanding of their symbioses and speciation.

Additionally, although *Ae. aegypti* is frequently artificially infected with *Wolbachia* for biocontrol purposes [105–109], our findings suggest that infected *Ae. aegypti* might not be stably maintained in the wild. This may be advantageous for vector population suppression as the cytoplasmic-incompatibility effect of any artificially introduced *Wolbachia* strain will likely be fully manifested in the uninfected native population [21]. However, this also implies that this type of biocontrol method may have low long-term effectiveness if the infection cannot be naturally sustained in the wild population. The detection of natural *Wolbachia* infection in wild *Ae. aegypti*, therefore, has huge implications for vector control programmes [21]. Not only does it inform the selection of a suitable *Wolbachia* strain prior to its field release, but it can also be used to gauge the long-term effectiveness of a specific vector control programme.

Interestingly, the sex of the mosquitoes had an effect on their *Wolbachia* infection status. This could be an artefact of various *Wolbachia*-induced reproductive phenotypes, such as parthenogenetic and male-killing ones, resulting in offspring that are largely female [15]. If this were true, over multiple generations with vertical *Wolbachia* transmission, one should observe an increasing proportion of females that are infected. Hence, the phenomenon observed here could be a consequence of reproductive manipulation by *Wolbachia* and vertical transmission.

While we were unable to statistically test for the effects of locality on infection status due to uneven and small sample sizes of the respective species across different localities, our results suggest that mosquitoes found in localities across Singapore have roughly equal chances of harbouring *Wolbachia*. This also suggests that underlying physiological factors and phylogenetic relatedness in mosquitoes contribute more to their infection by *Wolbachia* than the habitat in which they are found.

The reproductive effect of *Wolbachia* can be masked or enhanced by other reproductive endosymbionts such as *Cardinium*, *Rickettsia*, and *Spiroplasma* [7, 26–29]*.* Unfortunately, we were unable to detect these endosymbionts due to a high degree of false positives with the PCR-based screening methods used here (Additional file 1). This was likely due to using primers that are not optimised for screening mosquito-specific endosymbionts [110–112]. As a result, co-infections with various reproductive endosymbionts, which would have provided greater insights into the synergistic effects of co-infections on mosquito evolution, could not be identified among the wild mosquitoes examined here. There is, hence, a need to develop and optimise alternative screening methods, such as multilocus sequence typing (MLST) techniques, especially for the detection of *Cardinium*, *Rickettsia*, and *Spiroplasma* in mosquitoes.

### Tissue tropism of *Wolbachia* infection in mosquitoes

*Wolbachia* were detected mainly in the reproductive tissues, which agrees with results from studies across multiple insect groups [15, 84, 113], and suggests that *Wolbachia* are mainly vertically transmitted. Interestingly, through the course of this study, there was significant variation in reproductive traits (testis and ovary length) across and within species. These reproductive traits did not vary significantly with *Wolbachia* infection status, even after accounting for phylogenetic relatedness (see Additional file 2).

Infection in the gut and leg tissues was detected, albeit infrequently. This is not surprising, as previous studies have also detected *Wolbachia* in those tissues [34–36, 114]. Interestingly, the nucleotide sequences from gut and leg infections tend to be shorter in length. Considering that *Wolbachia* are unlikely to survive extracellularly for a long period of time [35], the small amplicon size suggests potential horizontal integration of the *Wolbachia* genome into the host genome for a few species. This phenomenon has been observed in several *Wolbachia* hosts [115, 116], and mosquito species such as *Ae. aegypti* and *Cx. quinquefasciatus* [117, 118]. A recent study showed that horizontal integration of the *Wolbachia* genome into the host genome can have implications for sex determination and evolution. This is evident in the common pillbug *Armadillidium vulgare*, and results in the formation of a new sex chromosome [119]. Researchers have also proposed that horizontal gene transfer between an endosymbiont and host can result in evolutionary innovation where new functional genes arise in both host and bacteria [117, 118].

Future research should explore the relative importance of each transmission method with relation to host-endosymbiont ecology and evolution. Tissue-specific screening methods such as those used here can be used in other arthropods, especially when the mode of transmission is not clear. Currently, most *Wolbachia* screening is conducted on ground specimens or specimens in their entirety [39–41]. In these cases, researchers are unable to determine tissue tropism of *Wolbachia* infection, which could provide clues to its mode of transmission. Thus, adopting tissue-specific screening methods would enable researchers to verify or refute the commonly reported assumption that *Wolbachia* is transmitted vertically [15, 30].

### Diversity and host-specificity of *Wolbachia* strains

Not only does the *wsp* molecular marker allow successful detection of *Wolbachia* infection across numerous taxa, it also enables strain genotyping and evolutionary comparison between detected *Wolbachia* strains [60]. In this study, *Wolbachia wsp* sequences were clustered into 12 putative *Wolbachia* strains falling within supergroup A or B. This is consistent with the results of previous studies that looked at *Wolbachia* infections in mosquitoes [39, 80, 85]. Each mosquito host species was only infected by strains belonging to supergroups A or B, with the exception of *Ae. albopictus*, which harboured both. Infection with more than one strain (superinfection of wild *Ae. albopictus* with *Wolbachia* supergroups A and B) has been previously reported, and this phenomenon was commonly observed to be fixed in the examined populations due to strong cytoplasmic incompatibility effects [120, 121]. This suggests stable vertical transmission of both strains in *Ae. albopictus*. Additionally, only four out of 12 putative strains were identified as previously typed *Wolbachia* strains reported by Zhou et al. [60] and Ruang-Areerate et al. [80]—Wol 1, Wol 2, Wol 3, and Wol 8 were identified as *w*Pip, *w*AlbB, *w*Cra, and *w*Ri strain, respectively.

Host specificity is thought to be a characteristic of the ancestral *Wolbachia* strain, with host flexibility reported mainly in *Wolbachia* supergroups A and B [122]. In our study, we found a combination of specialists and generalists, with more of the former. A study of mosquitoes from Taiwan showed a similar pattern [84]. In beetles, a mixture of *Wolbachia* supergroup A host-specific and host-flexible strains within a population has also been reported [49]. While our estimates of specialists and generalists might vary with greater sampling effort, the higher numbers of specialists observed can be explained by the process of reciprocal selection between host and endosymbiont over evolutionary time [123]. This is also known as Red Queen dynamics, where the endosymbiont constantly adapts to its host to ensure continued establishment in the same host [124]. An alternative, generalist strategy can also be maintained in a population. It ensures survival in an environment where resources (i.e. hosts) are rarely found [123]. However, there are generally more instances of host specialists than generalists across numerous parasitic and endosymbiotic taxa [125–127].

The SPS scores revealed that host flexibility among the generalists varied greatly. Understanding *Wolbachia* host specificity has huge implications, especially for the optimisation of *Wolbachia* biocontrol strategies. Not only should researchers select strains that can effectively limit pathogen replication [128], they should also select strains for their host specificity. This is not possible without the screening of a wide variety of species or closely related species, which was achieved in this study. Using a host-specific strain will decrease the likelihood of host shift to non-target species, and thereby minimise the overall ecological risk of a strategy.

### Evolutionary relationships between mosquitoes and *Wolbachia*

Host-*Wolbachia* relationships are often understudied and limited to a few taxa [52]. Studies have shown that the evolutionary associations between *Wolbachia* and their insect hosts do vary across taxa [49–52, 129]. Likewise, our exploratory analyses of mosquito hosts and their *Wolbachia* infection support such a complex relationship, with neither co-speciation nor host shifting fully accounting for evolutionary association in these lineages.

Based on the tanglegram, a broad association pattern between mosquitoes and *Wolbachia* strains was observed (Fig. 3). *Aedes* mosquitoes tended to be associated with *Wolbachia* supergroup A, while other mosquito species, particularly of the genus *Culex*, were largely associated with *Wolbachia* supergroup B. This showed that closely related *Wolbachia* strains are likely to establish themselves in related hosts. There might have been radiation of *Wolbachia* in these clades after their respective initial establishment. Nevertheless, the observed variations in host-endosymbiont associations make us question the mosquito-*Wolbachia* association pattern.

The ParaFit analysis showed weak support for congruency between host and endosymbiont phylogenies. Among the 18 host-*Wolbachia* associations, only the link between *Mansonia indiana* and Wol 3 showed a significant association (Fig. 3). This was interesting considering that Wol 3 was largely host flexible. Given that this was the only significant association, it is worth carrying out further genus-specific study on *Mansonia* spp. to elucidate coevolutionary patterns within a group of closely related mosquito species. It is possible that the degree to which *Wolbachia* co-evolve with their mosquito hosts varies across different taxonomic levels [74]. The analyses carried out thus far suggest that mosquito-*Wolbachia* associations are likely random at higher taxonomic levels, and that mosquito-*Wolbachia* co-speciation occurs at finer phylogenetic resolution (i.e. similar to patterns seen in diffuse coevolution).

The event-based analysis performed in Jane 4.0 (Fig. 4) indicated that co-speciation events were infrequent as compared to other evolutionary events. We noticed a greater proportion of host shifts and numerous losses. Interestingly, the least cost coevolutionary reconstruction indicated multiple consecutive host shifts occurring near the tips of the cladogram. This suggests that co-speciation does not fully explain the evolutionary associations between mosquito hosts and *Wolbachia*. Instead, recent host shifting through horizontal transmission seems to promote *Wolbachia* diversification. This lends greater support to the idea that horizontal transmission between distantly related species is possible [32, 33, 130].

Furthermore, losses, which represent endosymbiont extinction events that occurred upon host speciation, seem to dominate the evolutionary history of *Wolbachia*. Extinction events are believed to be frequent in host-endosymbiont systems [123], due to either evolution of resistance in the host or declining host population size, which result in the inability of highly specialised endosymbionts to establish themselves [131, 132]. Additionally, losses could potentially influence endosymbiont evolution through the creation of vacant niches [131]. The observed losses followed by host shifts in the mosquito-*Wolbachia* relationship are possible consequences of vacant niche exploitation by generalists. Perhaps this enabled successful endosymbiont invasion due to minimal intra-strain competition. If this were true, horizontal *Wolbachia* transmission and losses may play a bigger role in accounting for *Wolbachia* diversity than previously thought.

As this was an exploratory study, we were unable to determine the exact mechanism behind the diversity and evolutionary associations of *Wolbachia*. The presence of numerous specialists could be a sign of mosquito-*Wolbachia* coevolution since coevolution is fundamentally reciprocal selection between host and endosymbiont which gives rise to micro-evolutionary changes [133]. The numerous host shifts and losses might have, however, blurred the effects of vertical transmission over a long evolutionary period [52]. Thus, co-speciation might have occurred within smaller clades of *Wolbachia* and mosquitoes, but at higher taxa levels, horizontal transmission and loss events are more likely the prominent force driving *Wolbachia* evolution.

### Strengths, limitations, and future directions

The three distinct methods employed here to explore evolutionary associations have both strengths and limitations. The tanglegram allows for clear visualisation of host-endosymbiont association without taking into account any evolutionary relationships, but there have been calls for careful interpretation of the results generated using this method as the degree of entanglement may not necessarily represent phylogenetic congruence [134]. The Global ParaFit test seeks to address this limitation by testing for global congruency with an unbiased, statistical approach [74]. The event-based method enables the evaluation of potential evolutionary events that might have occurred throughout an endosymbiont’s evolutionary history such as co-speciation, duplication, and host shifting. This last method, however, cannot fully differentiate a topological congruence from an evolutionary event [135]. Without knowledge of the time of divergence for both symbiont and host, a co-phylogenetic pattern may be better explained by ecological factors (as compared to co-speciation) given that bacterial lineages often evolve faster than their hosts [136, 137], and the high likelihood of host shifts among closely related species [133].

The *Wolbachia wsp* gene has been shown to provide well-resolved phylogenies [60], and this study provides an exploratory snapshot of the evolutionary associations between mosquito hosts and their *Wolbachia* endosymbionts*.* There is, of course, a potential caveat, since only a single gene was used to construct the respective phylogenetic trees. To obtain a more accurate phylogeny, future studies could adopt MLST [17, 51], or whole-genome shotgun sequencing [52]. The former could potentially characterise putative *Wolbachia* strains that cannot be distinguished with *wsp* gene primers.

Notwithstanding their limitations, the employment of various analytical methods allows for a comprehensive examination of the evolutionary associations between *Wolbachia* and mosquito hosts which are presently lacking in the literature. The scope of future studies that examine the evolution of medically important vector species could be narrowed to the Aedini tribe, as this would provide greater statistical power for the examination of mosquito-endosymbiont associations.

## Conclusion

To our knowledge, this is the first study to examine *Wolbachia* infections in wild mosquitoes in Singapore. We detected 12 putative strains of *Wolbachia* among 40 mosquito species, and recorded infections in seven species for which, to our knowledge, *Wolbachia* infections have not been previously reported. By employing a tissue-specific PCR screening method, we were able to observe that the *Wolbachia* infections were preferentially located in the reproductive tissues, which provides support for vertical transmission as the main mode of infection transmission. However, even if *Wolbachia* infection is mainly transmitted vertically, this is unlikely to fully explain the observed diversity of *Wolbachia* and why closely related *Wolbachia* lineages were found in distantly related mosquito species. Hence, this study also served as an exploratory study which examined mosquito-*Wolbachia* evolutionary associations across a wide range of host mosquito species through three evolutionary analyses. Overall, we propose that the evolutionary associations between mosquito hosts and *Wolbachia* are consequences of both vertical and horizontal transmission and various evolutionary events.

## Supplementary information

**Additional file 1: Table S1.** Polymerase chain reaction (PCR) screening of *Cardinium*, *Rickettsia*, and *Spiroplasma* in wild mosquitoes from Singapore.

**Additional file 2: Figure S1.** Weighted reproductive tissue length across various mosquito species.

## Data Availability

The datasets generated and/or analysed during this study are available in the Dryad repository, https://doi.org/10.5061/dryad.zs7h44j63. Sequence data that support the findings of this study have been deposited in Genbank with the accession codes MT645167–MT645184.

## Abbreviations

BLAST: Basic Local Alignment Search Tool
*cox*1: Cytochrome c oxidase subunit I gene
MLST: Multilocus sequence typing
mtDNA: Mitochondrial DNA
NJ: Neighbour joining
PCR: Polymerase chain reaction
SPS: Standardised phylogenetic host specificity
*wsp*: *Wolbachia* surface protein gene
