# Supplementary material for: Wolbachia infection in wild mosquitoes (Diptera: Culicidae): implications for transmission modes and host-endosymbiont associations in Singapore
Source: Parasit Vectors. 2020 Dec 9;13:612. doi: 10.1186/s13071-020-04466-8 (PMC7724734; doi:10.1186/s13071-020-04466-8)
Supplement: Supplementary file 1 — Additional file 1: Table S1. Polymerase chain reaction (PCR) screening of Cardinium, Rickettsia, and Spiroplasma in wild mosquitoes from Singapore. [file 13071_2020_4466_MOESM1_ESM.docx]

**Additional file 2. Figure S1. Weighted reproductive tissue length across various mosquito species.**

**
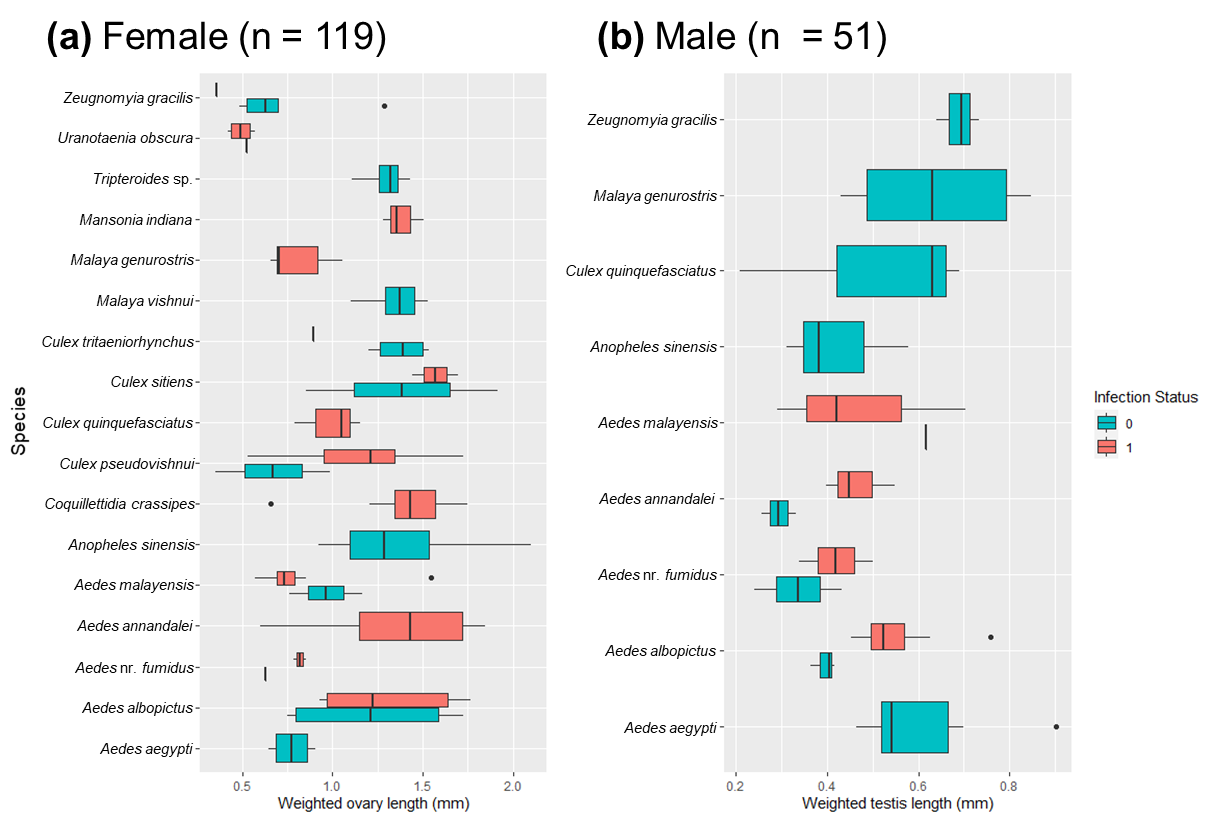
**

Weighted reproductive tissue length of (**a**) females (ovary), (**b**) males (testis). For females, the length of the ovary was obtained from the average of two readings for each of the ovary. Likewise, for males, the length of testis was obtained from the average of two readings. To account for body size differences, length of reproductive tissues was weighted against the mosquito individual’s thorax depth by dividing the raw measurement with the thorax depth. Blue represents non-infected individuals; red represents infected individuals. Bolded vertical lines indicate the median, error bars represent standard error, dots represent outliers. Overall, the figure showed reproductive trait variation across and within species. Additionally, phylogenetic independent contrast (PIC) was conducted to examine the correlation between infection prevalence and mean weighted reproductive tissue length of the species. PIC was conducted using the neighbour-joining *COI* tree generated from the study to account for phylogenetic effects. PIC showed that there was no correlation between the length of the reproductive trait and *Wolbachia* prevalence in mosquitoes (Phylogenetic independent contrast: female: *F*_(1,15)_ = 0.042, *P* = 0.839; male: *F*_(1,7)_ = 2.33, *P* = 0.171).
