# Supplementary material for: Wolbachia infection in wild mosquitoes (Diptera: Culicidae): implications for transmission modes and host-endosymbiont associations in Singapore
Source: Parasit Vectors. 2020 Dec 9;13:612. doi: 10.1186/s13071-020-04466-8 (PMC7724734; doi:10.1186/s13071-020-04466-8)
Supplement: Supplementary file 2 — Additional file 2: Figure S1. Weighted reproductive tissue length across various mosquito species. [file 13071_2020_4466_MOESM2_ESM.docx]

**Additional file 1. Table S1. PCR screening of *Cardinium*, *Rickettsia*, and *Spiroplasma* in wild mosquitoes from Singapore.**

| **Reproductive endosymbionts** | **Individuals screened**  **(Tissues screened)** | **Potential infection (%)** | **Amplicons sent for sequencing** | **Confirmed infection (%)** |
| --- | --- | --- | --- | --- |
| *Cardinium* | 126 (378) | 13/126  (10.3%) | 12 | 2/12 (16.7%) |
| *Rickettsia* | 126 (378) | 36/126  (28.6%) | 21 | 0/21 (0%) |
| *Spiroplasma* | 62 (186) | 55/62  (88.7%) | 16 | 0/16 (0%) |

The following primers were used for PCR: (i) *Cardinium* (16S rRNA) CarFB-F: GCGGTGTAAAATGAGCGTG and CarFB-R: ACCTMTTCTTAACTCAAGCCT [1]; (ii) *Rickettsia* (citrate gene) Rics741-F: CATCCGGAGCTAATGGTTTTGC and Rcit1197-R: CATTTCTTTCCATTGTGCCATC [2]; (iii) *Spiroplasma* (intergenic ribosomal spacer and adjacent regions between the 3’-end of 16S and 5’-end of the 23S) Spits-J04: GCCAGAAGTCAGTGTCCTAACCG and Spits-N55: ATTCCAAGGCATCCACCATACG [3]. Tissues screened refers to the total number of tissues screened for the endosymbiont. This number includes leg, gut, and reproductive tissues of each screened individual. Potential infection is calculated based on amplicon visualisation without additional DNA sequencing. Confirmed infection was determined after sending a subset of amplicons for Sanger sequencing. Results suggest that the percentage of true positives was very low for the screening of these endosymbionts. This implies that DNA sequencing as a follow-up procedure is necessary to validate infection especially for the screening of *Cardinium*, *Rickettsia*, and *Spiroplasma*.

**References**

1. Weeks AR, Velten R, Stouthamer R. Incidence of a new sex-ratio-distorting endosymbiotic bacterium among arthropods. Proc R Soc Lond B. 2003;270:1857–65.

2. Davis MJ, Ying Z, Brunner BR, Pantoja A, Ferwerda FH. Rickettsial relative associated with papaya bunchy top disease. Curr Microbiol. 1998;36:80–4.

3. Majerus TMO, Graf Von Der Schulenburg JH, Majerus MEN, Hurst GDD. Molecular identification of a male-killing agent in the ladybird *Harmonia axyridis* (Pallas) (Coleoptera: Coccinellidae). Insect Mol Biol. 1999;8:551–5.
